# Evaluating the basis for use of advanced stage as a surrogate endpoint for cancer mortality in screening trials: a simulation study of meta-correlation and an alternative framework

**DOI:** 10.1016/j.eclinm.2026.103862

**Published:** 2026-04-01

**Authors:** Peter D. Sasieni, Adam R. Brentnall, Stephen W. Duffy

**Affiliations:** a Centre for Cancer Screening, Prevention and Early Diagnosis, Wolfson Institute of Population Health, Queen Mary University of London, London, UK; b Centre for Evaluation and Methods, Wolfson Institute of Population Health, Queen Mary University of London, London, UK

**Keywords:** Surrogate endpoints, Cancer screening, Meta-analysis, Causal inference, Advanced stage, Simulations

## Abstract

**Background:**

Surrogate endpoints have been proposed to accelerate the evaluation of cancer screening but are controversial. Recent systematic reviews of advanced stage and cancer mortality in randomised trials of cancer screening have reached different conclusions regarding the suitability of advanced stage as a surrogate endpoint. We aimed to consider the suitability of trial-level meta-analysis to evaluate surrogacy and propose an alternative practical framework to evaluate potential surrogate endpoints for cancer screening trials.

**Methods:**

In this simulation study, we investigated whether a potential surrogate endpoint (advanced stage) could be used in place of cancer-specific mortality in a randomised controlled trial of cancer screening versus no screening. We focused on types of cancer screening that lead to the early detection of invasive cancer. We used in silico experiments to study meta-analysis correlation when the data are generated using a valid surrogate endpoint (one that mediates the effect of screening on mortality) or an invalid surrogate endpoint (one affected by screening without screening altering mortality). Simulations of the three surrogate scenarios considered stage-specific incidence and prognosis based on cancers of the breast, bowel, liver, lung, ovary, and prostate. Parameters in the simulations were based on stage-specific incidence, prognosis, and trial size were based on the trials included in the systematic review of Feng et al. (2024, 39 trials; source data https://github.com/hilaryrobbins/cancer_screening_endpoints); except for breast cancer. Population inclusion criteria and most parameter values in the simulations were taken from a systematic review of cancer screening trials. Alternative tests of necessary conditions for late-stage cancer incidence to be an accurate surrogate endpoint were developed.

**Findings:**

Trial-level correlation between the stage and mortality endpoints may be weak even when stage fully mediates the effect of screening on mortality. In the simulations for all cancers combined, if advanced staged mediated 100% of the screening effect and screening was effective then correlation was 0.61. Further, an invalid surrogate can also have a substantial correlation. In the simulations, if advanced stage mediated none of the screening effect the correlation across all cancers was 0.41. A series of ancillary studies to establish the utility of a surrogate endpoint will provide stronger and more timely evidence to evaluate surrogate endpoints than trial-level correlation; supported by further studies to establish a mortality benefit.

**Interpretation:**

Meta-correlation is not a reliable metric for assessing the adequacy of a surrogate endpoint for cancer screening interventions. A surrogate should be backed by causal reasoning rather than meta-analysis. To accelerate evidence generation, trials using a good candidate surrogate endpoint should be conducted in parallel with other studies to test the suitability of the endpoint. Future work is needed to determine acceptable criteria to validate cancer screening surrogate endpoints using evidence from such studies.

**Funding:**

10.13039/501100000289Cancer Research UK.

**Research in context**

**Evidence before this study**

The literature recommends meta-analysing the results of multiple randomised clinical trials to determine whether a putative surrogate endpoint is an adequate surrogate. We previously co-authored a systematic review and meta-analysis of advanced stage as a surrogate endpoint for cancer screening interventions, that followed two other systematic reviews and meta-analysis on this topic. Subsequently, we considered citations from and of these systematic reviews using Google Scholar in July 2025, and again in January 2026. This identified that three systematic reviews have used meta-correlation to assess the adequacy of advanced stage as a surrogate endpoint for cancer mortality across multiple classes of screening test and cancer sites. They came to very different conclusions. To address this inconsistency within the literature, we aimed to investigate this further and evaluate two methodological questions: (1) Is trial-level correlation a reliable method for determining whether an endpoint is a useful surrogate for cancer-screening trials? (2) What testable assumptions or conditions ensure that late-stage cancer incidence is an accurate surrogate endpoint?

**Added value of this study**

Our findings show that meta-correlation lacks empirical validity. In response, we offer an alternative strategy for assessing incidence-based surrogates for cancer screening. This uses a series of studies to be done prior, alongside, and after using an unproven surrogate endpoint to enable valid inference regarding cancer mortality in cancer screening trials.

**Implications of all the available evidence**

Given that this work shows meta-correlation to be a poor metric for evaluating surrogate endpoints, further meta-analysis of surrogate endpoint in cancer screening is unlikely to be useful. For any new type of screening, by the time there are enough trials to robustly meta-analyse, there will also be enough trials to determine the impact of screening on cancer mortality. Accelerating progress in evaluating the clinical utility of new cancer screening modalities requires causal reasoning and evidence from randomised controlled trials and observational studies.

## Introduction

Surrogate endpoints for cancer screening trials would, if accepted, enable faster, more efficient research. Regulators require surrogate endpoints used in therapeutic trials to pass at least two tests. First, they should be associated with the gold standard outcome in cohorts with no intervention (individual-level surrogacy). For cancer screening trials, cancer stage is an individual-level surrogate because it is highly prognostic of mortality. Second, they should enable inference about the effectiveness of an intervention, i.e., the effect of screening on the surrogate should predict its effect on the target endpoint (trial-level surrogacy). For example, if the surrogate indicates superiority in the intervention arm, one may infer that the intervention is effective. The extent to which advanced stage is a trial-level surrogate is a more controversial issue, and a hot topic partly due to a screening trial that has advanced stage incidence as its primary endpoint (primary analysis expected 2026).^1^

This paper is focused on types of cancer screening that lead to the early detection of invasive cancer. Different considerations would be relevant for screening that primarily prevents cancers by diagnosing (and then treating) pre-cancerous lesions.

Four reviews on this issue have been published since mid-2024.^2^, ^3^, ^4^, ^5^ One, a narrative review, stated: “The strongest evidence comes from synthesis of randomised controlled trials with meta-regression”.^2^ Much of this evidence was synthesised for advanced stage by the other three papers.^3^, ^4^, ^5^ All three evaluated surrogacy primarily based on trial-level correlation between advanced cancer incidence and cancer-specific mortality. Despite having broadly similar objectives, and using similar methodology, the conclusions drawn were quite different. The authors of the most recent systematic review^5^ reflected on potential reasons. These included discussion on important differences in trials included (Feng et al.^4^ included n = 39 trials, Dai et al.^3^ n = 33, and Rebolj et al.^5^ n = 57), and the role of timing of different endpoints within the included trials, and the definition of advanced stage.

Motivated by the analysis and findings from these reviews, we aimed to evaluate two methodological questions: (1) Is trial-level correlation a reliable method for determining whether an endpoint is a useful surrogate for cancer-screening trials? (2) What testable assumptions/conditions ensure that late-stage cancer incidence is an accurate surrogate endpoint?

To address the first question, we conducted in silico experiments to examine results from trial-level correlation analysis of a so-called perfect surrogate outcome (one that mediates all the effect of screening), and from scenarios in which screening reduces advanced stage cancer but does not impact on cancer mortality. The simulations used scenarios based on data from the systematic reviews (including sample sizes). We also used the simulation study to consider whether meta-regression may be preferrable to meta-correlation.

To address the second question, we first considered methods available and commonly used to justify surrogate endpoints to regulators.^6^^,^^7^ We noted that some approaches such as the Prentice criteria,^8^ or Freedman measures,^9^ are not often used due to practical or methodological issues, such as conditioning on a post-randomisation variable. Multivariate meta-analysis of summary data is a commonly applied method and another focus in the literature is evaluating the surrogacy of causal effects (e.g. the effect of screening on those who receive it). Therefore, we proceeded to consider if meta-analysis is insufficient from a causal perspective to evaluate surrogacy, by considering how an endpoint that is proportional to the reduction in mortality in one set of trials, could be useless as a surrogate in a new trial.

Establishing trial-level surrogacy is always difficult. It is particularly so in the field of cancer screening because there are so few adequately powered trials of any one type of screening. A key difference between cancer screening and therapeutic trials is the scale and costs required to evaluate mortality. This had led to there being relatively few cancer screening trials. This makes application of most of the existing methods to evaluate surrogacy impractical for new classes of screening test, even if only a single trial is used^9^ since that trial needs to include the primary mortality endpoint, and if successful it is likely to be inefficient to run another trial of the same technology. Systematic reviews of the field suggest that there have been fewer than 60 randomised controlled trials comparing any type of cancer screening to no screening that have published results both on cancer mortality and a surrogate endpoint such as cancer stage at diagnosis. Many of these trials are old and it is not possible to get hold of the individual level data that is required for some of the more detailed analyses of surrogacy. To proceed we therefore took a stance that, a priori, advanced stage is a reasonable candidate for a surrogate endpoint and aimed to develop a practical framework to help answer the question: What might go wrong when using advanced stage instead of mortality? To tackle this, we developed a strategy to assess the usefulness of advanced stage as a potential surrogate endpoint for cancer-screening tests other than those previously trialled.

## Methods

### Study overview and data sources

We first conducted a computer simulation study to evaluate the expected correlation and linear regression from trial-level meta-analysis when using advanced stage as a surrogate endpoint. Simulations of the three surrogate scenarios considered stage-specific incidence and prognosis loosely based on breast, bowel, liver, lung, ovary, and prostate cancers (Table 1). Parameters in the simulations were based on stage-specific incidence, prognosis, and trial size were based on the trials included in the systematic review of Feng et al. (2024, 39 trials; source data https://github.com/hilaryrobbins/cancer_screening_endpoints); except for breast cancer. The reason for the breast cancer parameters was that the proportion of stage I cancer diagnosed symptomatically has increased since the trials. We then considered alternative framework for evaluating advanced stage as a surrogate.

**Table 1:** Parameters used in the simulations.

Site	Number per arm (1000s)	Proportion of population with advanced stage (P_a_)	Proportion of population with early stage (P_e_)	Fatality of advanced stage (F_a_)	Fatality of early stage (F_e_)
Breast	18–80	0.4–1.0%	2P_a_	40%	10%
Bowel	14–85	0.5–1.1%	P_a_	65%	20%
Lung	1–27; 65–80^a^	0.7–3.3%	0.5P_a_	90%	10%
Ovary	10–155	0.4–1.0%	0.4P_a_	70%	25%
Prostate	7.5–220	0.8–3.5%	3P_a_	28%	4%
Prostate with short follow-up	9–225	0.5–2.0%	4P_a_	10%	1.5%
Liver	2–25	0.4–2.5%	0.75P_a_	95%	85%

The parameter choices outlined here are based on the trials in Feng et al. (2024, 39 trials).

a 15% of trials are small (N∼U (900, 27,000)) and 85% are large (N∼U (65,000, 80,000))

### Ethics

Ethics approval was not required because there were no new human study subjects for this paper and all analyses of human data used on publicly available summary statistics. For the same reasons there was no requirement for written informed consent.

### Data analysis

Trial-level correlation and regression was investigated under three simulation scenarios.

1. Screening is effective in reducing mortality and the surrogate is valid.
2. The null hypothesis holds, screening is ineffective, and the surrogate is valid.
3. Screening is ineffective on mortality but reduces the surrogate endpoint, so the surrogate is invalid.

For simplicity, in the simulations we considered the endpoints as binary because in most cancer screening trials the events (death from the targeted cancer or diagnosis with advanced stage cancer) are rare so the proportions test is efficient for the logrank test.^10^ Although that means that results are hardly changed if we consider the rate of events in each arm or the proportion of participants in each arm with an event, it does prevent us from exploring some of the key features of the data evolution. In practice cancer diagnoses occur before cancer deaths and individuals could be censored between diagnosis and death. This is more likely in the screening arm since screen-detected cancers will have been diagnosed earlier than they would otherwise have been. It also ignores the fact that the distribution of the events (cancer deaths and advanced stage diagnoses) is unlikely to be exponential and the hazard ratios between the two arms is unlikely to be constant over time. Binary endpoints also gloss over the complexities of lead time. Lead time effects survival (and fatality) if measured from diagnosis but not if measured from randomisation. Thinking about the events as they develop over time makes it clear that any endpoint based on characteristics of cancers at diagnosis needs to follow-up participants for at least one screening interval after the last screen to allow time for cancers that would have been screen-detected in the screening arm to be diagnosed symptomatically in the control arm.

We next introduce notation needed to fully describe the assumptions of the three scenarios. Let the expected proportion of randomised participants in the control arm with advanced cancer be P_a_ and the proportion with early cancer P_e_. Let the expected proportion with advanced cancer in the screening arm be θP_a_, so that θ is the relative risk of advanced cancer. Assuming the screening test does not lead to cancer prevention, the expected proportion in the screening arm with a true (i.e., not over-diagnosed) early cancer is P_e_ + (1 – θ)P_a_. Next, let ψ denote the relative risk of cancer-specific mortality; F_a_ the probability that a patient diagnosed with advanced cancer will die from their cancer, and F_e_ the corresponding probability for early-stage cancer. For the valid surrogacy scenario 1, we assume that these probabilities are not affected by screening, i.e., cancers diagnosed at advanced stage have fatality F_a_ and cancers diagnosed at early stage have fatality F_e_, regardless of whether the cancer was detected by screening or not. In the case of overdiagnosis (i.e. screening leads to diagnosis of a cancer that would not otherwise have been diagnosed in the individual's lifetime), F_e_ in the screening arm is the probability of an individual with a ‘not over-diagnosed’ early cancer dying from their cancer (since, by definition, the probability of dying from an over-diagnosed cancer is zero). We note that some overdiagnosed cancers might have proved fatal had the patients not died earlier from a competing cause. In that sense we are treating the fatalities as absolute rather than net.

We use ‘fatality’ when the denominator is people diagnosed with cancer, and ‘mortality’ when the denominator is the whole cohort.

Using this notation, we define the three scenarios as:

1. ψ < 1, and ψ = {θP_a_F_a_ + [P_e_ + (1 – θ)P_a_]F_e_}/{P_a_F_a_ + P_e_F_e_};
2. ψ = 1, and ψ = {θP_a_F_a_ + [P_e_ + (1 – θ)P_a_]F_e_}/{P_a_F_a_ + P_e_F_e_}; and
3. ψ = 1, but θ < 1.

To explore what happens when the chosen endpoint is an invalid surrogate, we assume that screening causes some tumours which would have been advanced stage at diagnosis to be detected at early stage, but these cancers retain the same fatality as if they had been diagnosed at advanced stage. In this scenario, stage still predicts fatality in all those cancers not down-staged by screening, but the expected proportion of all (not over-diagnosed) cancer patients dying is the same in both arms regardless of the value of θ. Thus, screening prevents advanced stage cancers, but it has no effect on cancer mortality.

Under the assumption of fatality being independent of screening conditional on the surrogate endpoint, and assuming F_a_ > F_e_ and P_a_ > 0, the cancer mortality ratio is less than one if and only if θ < 1. That is why we say it is a valid surrogate. In this model, the whole of the effect of screening on cancer mortality is via its effect on advanced stage. In the language of causal inference, advanced stage is a complete mediator: there is no effect of screening on cancer mortality other than that conferred by the effect on stage; and there is no effect of screening on stage that does not alter mortality.

We do not explicitly consider the case of partial mediation. This might arise for instance if the fatality of a screen-detected early-stage cancer is better than that of an advanced stage cancer, but not as good as a symptomatically detected early-stage cancer.

The simulations were designed to provide insight, rather than accurate predictions for each cancer site and test. For this reason, we also do not precisely define “advanced cancer”; we simply assume that in any simulated trial it is was well defined. Note also that modern treatments mean that fatalities for many cancers today are less than in Table 1. Sensitivity analyses considered using slightly different proportions of early-stage breast cancer, and fatalities chosen to reflect contemporary survival data as published, for instance, by Cancer Research UK and the American Cancer Society.

For prostate screening trials we considered scenarios with shorter and longer follow-up periods. This was because several trials^11^^,^^12^ have observed a greater impact on prostate cancer mortality with 12–16 years of follow-up than with 7–10 years of follow-up (due to a long lead-time). Simulations were further conducted under the null hypothesis (θ = 1) and under a plausible range of values of θ (allowing for some trials having an attenuated θ due, for instance, to non-compliance or contamination). Under the alternative hypotheses (θ < 1), we simulated results both under the valid surrogate model (invariance of stage-specific fatality) and for an invalid surrogate (no impact on cancer mortality despite impact on advanced stage). We used a variety of sample sizes (always with equal numbers in the two trial arms). These were chosen to correspond approximately to the trials in the published systematic reviews.

For each simulated trial and for each endpoint (mortality and advanced stage), we performed a two-proportion Z-test (without a continuity correction) between the two arms. The proportion of trials for which the test was significant (two-sided P-value <0.05 based on normal approximation) was recorded as the statistical power. We also evaluated the one-sided size (i.e. how often the null hypothesis was rejected in favour of screening when there was no effect on mortality), by determining the proportion of trials with “significant” results.

Correlation coefficients were based on the logarithm of the relative risk for mortality and the logarithm of the relative risk for advanced stage. Following Feng et al. (2024), we use Pearson (unweighted) correlation, but in the Supplement, we repeat the analysis using weighted correlations with weights inversely proportional to the variance of the logarithm of the relative mortality (sensitivity analysis). For regression, we estimate the estimated intercept and slope and their variances using ordinary least squares. Again, we present unweighted results in the main paper and weighted sensitivity analysis in the Supplement. We calculated the upper limit of the 95% confidence interval (CI) for the predicted expected relative mortality conditional on the relative risk of advanced cancer being 0.85 using the formula:

$$\exp\left\{\hat{\alpha }+\hat{\beta }\ln\left(0.85\right)+z_{0.975}\sqrt{\left(\;{\hat{\sigma }}_{\alpha }^{2}+2\;{\hat{\sigma }}_{\alpha ,\beta }\;\ln\left(0.85\right)+{\hat{\sigma }}_{\beta }^{2}\;{\ln\left(0.85\right)}^{2}\right)}\right\}$$

The value 0.85 is arbitrary. It was chosen because few trials are powered for a screening effect of less than a 15% reduction in cancer specific mortality and because at a relative risk of 0.85 for advanced stage, several single cancer meta-analyses have a 95% confidence interval (CI) for the effect of mortality that excludes 1.

We used the confidence interval rather than the prediction interval because we were not interested in the result in a particular trial but the underlying relationship between the effect on mortality and the effect on advanced stage. All simulations were performed in Stata (version 18). Code is provided in the Supplement S1.

In order to test necessary conditions for late-stage cancer incidence to be an accurate surrogate endpoint, we first considered more general circumstances when meta-analysis could be insufficient by considering the endpoint ‘screening uptake in the screening arm’. We studied the relationship between this endpoint and the relative reduction in cancer mortality in a set of reasonably homogeneous trials if compliance with randomisation is independent of the underlying risk of cancer mortality. We then considered a new trial with excellent compliance with randomisation but enormous delays in diagnosing cancer in screen positive individuals.

This analysis highlighted the importance of causal reasoning in evaluating surrogate endpoints and when considering how to use potential surrogate endpoints to enable faster robust decision making on screening programme implementation. We next considered what evidence should be required before using the potential surrogate endpoint in a trial, so that it may be reported earlier than cancer-specific mortality. In such a trial, the surrogate might be a co-primary endpoint with the gold-standard reported later, or the sole primary endpoint because, even with longer follow-up, the trial would be under-powered for cancer mortality. In either case, we required that the randomised-controlled trial (RCT) plans for and reports on longer follow-up than used for the primary publication of the surrogate endpoint. If the RCT shows that screening has a significant beneficial effect on the surrogate endpoint, and if preliminary health economic modelling suggests that the balance of benefits, harms and costs is favourable, then we believe that a pilot implementation study would be justified and could begin prior to trial mortality results. In this context, to further test the suitability of the surrogate, we examined what causal assumptions underlying the validity of the surrogate endpoint could be stress-tested using data from the trial, the pilot implementation study, and ancillary studies.

### Role of the funding source

The funders had no involvement in study design, data collection, data analyses, data interpretation, or the writing of the report.

## Results

### In silico experiments

Table 2 presents the average power to detect a statistically significant effect at a 5% level for cancer-specific mortality and advanced stage endpoints in the simulations. In all scenarios there was greater power for advanced stage. In the scenarios considered overall power was 39% greater for the surrogate (76%) than for mortality (37%). Hence, with a valid surrogate there will be trials where the surrogate endpoint yields a statistically significant result, and the true endpoint does not. Practically, this means a single trial where a significant result is observed on the surrogate (but not on mortality) cannot be used to conclude that a surrogate is invalid, as has sometimes been the interpretation. For instance, it is often said that the United Kingdom Collaborative Trial of Ovarian Cancer Screening (UKCTOCS)^13^ proves that advanced cancer is a poor surrogate because a significant reduction in stage IV cancer incidence was observed (albeit based on a post hoc analysis), but no reduction in ovarian cancer mortality. The results also emphasise the importance of better understanding of surrogate endpoints because of their potential to enable smaller (and shorter duration) clinical trials. Table 2 also presents that the empirical one-sided Type I error (size)—the proportion of simulation under the null in which the null hypothesis was rejected in favour of screening. It closely approximated the nominal value (2.5%) for both endpoints. If anything, use of advanced stage was conservative.

**Table 2:** Average power and size obtained using the mortality and the advanced stage endpoints from simulations by cancer site.

Cancer site	Power–mortality endpoint	Power–advanced stage endpoint	Size (one-sided)–mortality endpoint	Size (one-sided)–advanced stage
Bowel	38%	76%	2.4%	2.2%
Breast	24%	74%	2.5%	2.4%
Liver	4%	56%	2.6%	2.4%
Lung	58%	68%	2.3%	2.5%
Ovary	47%	80%	2.5%	2.3%
Prostate	68%	94%	2.4%	2.4%
Prostate- short follow-up	27%	90%	2.6%	2.2%
All combined	37%	76%	2.4%	2.4%

Power and size to obtain a significant result with a (two-sided) nominal significance level of 5%. We present the proportion of simulations that rejected the null hypothesis in favour of screening. The power is based on scenarios in which screening reduces cancer-specific mortality. “Size” is the empirical probability of rejecting the null hypothesis in favour of screening when the null hypothesis is true, i.e. for scenarios in which screening has no effect on cancer mortality nor on advanced stage, and should equal 2.5%.

Table 3 shows trial-level correlations between the screening effects on the surrogate and true endpoints. Although the correlation tended to be strongest in the case of a valid surrogate, substantial correlations were sometimes observed when the surrogate was invalid (notably when survival and prevalence of advanced stage were set based on cancers of the liver and lung). Thus, a strong trial-level correlation between the surrogate and true endpoint was not sufficient to conclude that the surrogate is valid. Conversely, a weak correlation did not mean that the surrogate was invalid. Provided the surrogate was correlated with mortality in the absence of screening, the relative risks were correlated even if it was not a valid surrogate. When restricted to smaller trials (N ≤ 25,000 per arm) correlations for valid surrogates were slightly smaller whereas those for invalid surrogates were larger (Supplementary Table S1). Using weighted correlation with weights inversely proportional to the variance of the logarithm of the mortality ratio, yielded slighted larger values for a valid surrogate and slightly lower for an invalid surrogate compared with the unweighted correlations (Supplementary Table S2).

**Table 3:** Average correlation of valid and invalid surrogate endpoints with mortality by cancer site in the simulation scenarios.

Cancer site	Valid surrogate		Invalid surrogate
	Correlation if screening is effective	Correlation under null hypothesis	
Bowel	0.75	0.69	0.44
Breast	0.58	0.51	0.32
Liver	0.60	0.75	0.58
Lung	0.93	0.92	0.67
Ovary	0.79	0.77	0.47
Prostate	0.70	0.44	0.17
Prostate- short follow-up	0.38	0.25	0.12
All combined	0.61	0.63	0.41

Table 4 gives results of the simulations with respect to the upper limit of the 95% CI when the surrogate relative risk is 0.85 (a reduction in the unfavourable outcome). For a valid surrogate, where there is a real effect on mortality, the confidence intervals indicate an effect on mortality is likely in the correct direction, except for the liver cancer scenario, where the CI for predicted mortality when the relative risk for the surrogate is 0.85, extended to 1.03. Conversely, for invalid surrogates, the expected upper limits corresponding to a surrogate relative risk of 0.85 were all greater than 1.00. So, in contrast to correlation which can be misleading, the fitted effect on mortality corresponding to a 15% reduction in advanced stage provided a warning of the unsuitability of the surrogate. The use of weighted linear regression (with weights inversely proportional to the variance of the logarithm of the mortality ratio) hardly changed the results (Supplementary Table S3).

**Table 4:** Average upper limit of 95% confidence interval (CI) on risk ratio (RR) for mortality if the risk ratio for the surrogate is 0.85.

Cancer site	Valid surrogate	Invalid surrogate
Bowel	0.93	1.03
Breast	0.94	1.03
Liver	1.03	1.04
Lung	0.88	1.05
Ovary	0.93	1.03
Prostate	0.91	1.03
Prostate- short F–U	0.92	1.02
All	0.93	1.03

Results from the simulated scenarios.

### Testing surrogacy conditions using complementary studies

Although the meta-regression line appears more informative than correlation coefficient, the following thought experiment demonstrates why meta-analysis is insufficient to evaluate potential surrogate endpoints to evaluate screening interventions. Suppose screening reduces cancer mortality by α = 1ψ in screened individuals. Suppose also that a proportion s_1_ are screened in the screening arm, (no individuals are screened in the control arm), and that whether an individual is screened is independent of their underlying risk of dying from cancer. Then the relative risk of cancer specific mortality is (ψs_1_ + 1−s_1_). So, mortality in the screening arm is reduced by s_1_α. Thus, the mortality reduction is directly linearly related to the screening uptake. Hence, if there are several large trials with varying levels of screening uptake (in the screening arm and no contamination in the control arm) that are otherwise homogeneous, we anticipate excellent meta-correlation with a meta-regression line with zero intercept.

Now consider a new trial using the same primary screening test, in which screening uptake is excellent. However, in the new trial, health services are unable to cope with the positive screening tests. Many individuals are never informed of their screening test result, and even when they are it takes months before they receive an appointment for diagnostic work-up. As a result, no one is screen-detected before they become symptomatic, and screening has no impact on cancer mortality.

In this example, empirical evidence from meta-analysis strongly supports ‘uptake’ as a surrogate endpoint, even though the most basic causal reasoning would warn against it. Although uptake mediates the effect of screening on mortality, uptake is not sufficient for screening to be effective.

The causal argument for using advanced stage is straightforward. Stage at cancer diagnosis is highly predictive of fatality. The intended mechanism of action of cancer screening is that it leads to earlier diagnosis of cancer. If the lead time is sufficient, the cancer will be diagnosed at an earlier stage than it would have been. If screening reduces mortality, it will do so (primarily) because those down-staged cancers will have better prognosis than they would have had had they not been down-staged. Thus, a priori, advanced stage is a good surrogate. But what might go wrong?

**Fig. 1:**
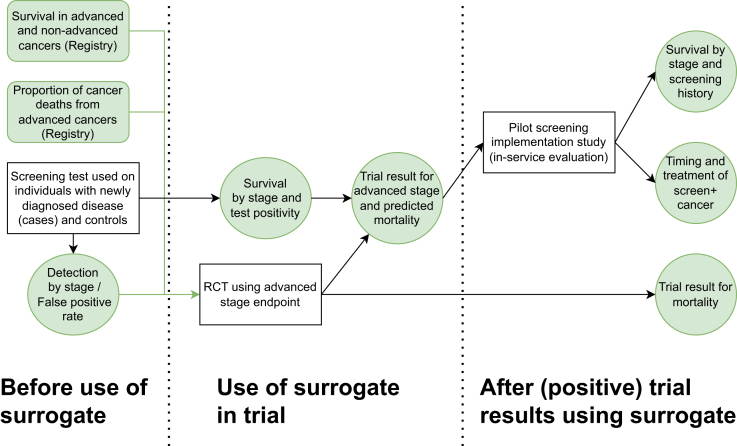
**Study design schematic**. Schematic of different study designs and how they might be used over time (in parallel and in series) to address both whether advanced stage is a reasonable surrogate for cancer mortality and whether screening has clinical benefit. Rounded boxes are studies of cancer survival in unscreened populations; square boxed are studies of cancer screening; and circles are outcomes of studies.

Fig. 1 shows how one might conduct a series of studies to test conditions necessary for surrogacy using different evidence strands. The ancillary studies to determine the reasonableness of advanced stage as a surrogate endpoint for a cancer screening trial or for use in modelling are described fully in Table 5. To illustrate how these might be used to evaluate surrogate endpoints, we also provide tentative “go/no-go” criteria for proceeding to the next level of testing (noting that other criteria might also be justified). These go/no-go thresholds are arbitrary (as is the traditional P < 0.05) and may be modified. They are presented to stimulate debate and structure thinking. They are not intended to determine regulatory decision making. The series of conditions proposed to be tested are as follows.

**Table 5:** Proposed steps in the verification of a potential surrogate endpoint.

Verification	Reason	Explanation	Heuristic Go/No-go criteria
**1 Stage-specific survival** Estimate stage-specific net survival from cancer registry data.	If there is no difference in survival between advanced stage and early-stage disease, then it is a poor surrogate	There is no mortality benefit of diagnosing at an earlier stage if the prognosis is the same for patients diagnosed at an earlier stage compared with those diagnosed with advanced cancer. More generally, the bigger the difference in survival between early stage and late-stage cancer the bigger the impact will be on mortality.	Go: Either (i) difference in 5-year net fatality between advanced stage and not advanced stage ≥30%; or (ii) difference ≥10% and relative 5-year net fatality ≥2 No-go: Both difference <10% and ratio <2.
**2 Stage-specific attributable mortality** Estimate the proportion of cancer deaths that originate from advanced stage cancers. Use Registry data.	If only a small proportion of cancer deaths originate from advanced stage cancers, then even eliminating them all would have little impact on cancer mortality	If advanced stage cancers are already rare then screening to prevent them will at most have a modest public health impact. Technically, this does not mean that advanced stage is not a surrogate–it is just not a very useful one.	Go: Proportion of cancer deaths (within 5-years of diagnosis) from advanced stage ≥50% No-go: Proportion within 5-years and within 10-years both <25%.
**3 Prognostic significance of screen-detectability** Use the screening test at the time of diagnosis in an unscreened cohort and follow for survival	To explore whether stage-specific survival depends on the cancer being screen-detectable	If screening preferentially finds cancers for which prognosis is poor no matter in what stage they are diagnosed, then there may be little or no benefit from screen-detection at an earlier stage. But if survival is independent of whether they are screen-detectable this is not an issue.	Go: For each stage, rate of death (with at least 1-year follow-up) in screen-detectable is <1.2 times rate in not screen-detectable and not no-go No-go: Average rate in screen-detectable early stage is no better than in screen-detectable advanced stage
**4 Predicted mortality** If stage-specific survival differs between screen-detectable and non-screen-detectable cancers, combine that survival with the stage of diagnosis of cancers in the randomised-controlled trial (RCT) to obtain predicted mortality that uses the differential survival between screen-detectable and screen non-detectable cancers.	To predict mortality based both on stage at diagnosis and on “detectability” (via the screening test)	Provides a more robust prediction of cancer mortality in the trial	Go: Either advanced stage or predicted mortality (from RCT or meta-analysis of RCTs) is significant at P < 0.05 (2-sided) and both are significant at P < 0.2 (1-sided) in favour of screening (corresponding to an 80% chance of not making a Type I error). No-go: 95% confidence interval (CI) for reduction in surrogate excludes any reduction; or 95% CI for reduction in surrogate excludes reduction for which trial was powered and 95% CI for predicted mortality excludes any reduction
**5 Survival by stage and screening history** Compare survival between screen-detected cancers, interval cancers, and cancers in individuals never screened in a large population. Ideally the ever-screened group should be split between “never offered screening” and “offered but not screened”.	To see if it is necessary to distinguish between screen-detectability at screening and screen-detectability at symptomatic diagnosis in terms of stage-specific survival	In the trial one only has screen-detectability at the time of screening. Using real-world data has the disadvantage that there may be underlying differences affecting survival between individuals who choose to go for screening and those who are never screened. Hence the need to distinguish between “Not invited” and “Invited but not screened”.	Go: Modelled effect (point estimate) on predicted mortality (taking into account differential survival) based on stage-specific sensitivities (and a natural history model) leads to a clinically meaningful reduction in cancer mortality. No-go: Even best-case scenario excludes clinically meaningful reduction in mortality
**6 Time from screening to treatment** Monitor time from screening to diagnosis and from diagnosis to initiation of treatment.	Concern that there may be a delay in initiating treatment of screen-detected cancers	It is possible, but hopefully extremely unlikely, that despite being diagnosed earlier (and at an earlier stage), individuals with screen-detected cancers face a delay in receiving treatment such that by the time they are treated they have already progressed to a more advanced stage.	No formal criteria for assessing surrogacy, but, for clinical audit, performance standards should be set
**7 Treatment of screen-detected cancers** Monitor the treatment received in a screening programme	Inferior treatment of screen-detected cancers would diminish the benefit of screening	It is possible that screening could be provided free whereas treatment is provided privately and that some patients with screen-detected cancers are unable to afford the best quality treatment. This should never happen within an RCT.	No formal criteria for assessing surrogacy, but, for clinical audit, performance standards should be set

Suggested verification steps to explore issues that could lead to advanced stage being inappropriate as a surrogate endpoint for cancer specific mortality on a cancer screening study. The Go/No-go criteria are provided to illustrate how data from each verification step could be used to evaluate whether to consider advanced stage as a reliable surrogate endpoint. They should not be thought of as hard and fast criteria to be adopted by decision makers.

Firstly, as proposed by Dai et al.,^3^ one should check that (1) there is a differential in survival between advanced stage and early-stage cancers of the type targeted by screening; and (2) that a substantial proportion of death from the targeted cancer originates from advanced cancers. These checks should be done before considering use of advanced stage as a surrogate endpoint. The second level of studies relate to using the screening test at the time of diagnosis and studying survival thereafter. Question (3) is whether the “screen-detectability” of the cancer at diagnosis affects prognosis. In this setting, one should compare (i) survival between early stage and advanced stage screen-detectable cancers and (ii) stage-specific survival between screen-non-detectable and screen-detectable cancers. If there is no survival advantage of early stage among screen-detectable cancer, it is unlikely to be a good surrogate. If there is a difference in stage-specific survival between screen-detectable and screen-non-detectable cancers, then one should plan to use predicted mortality taking account of the differential survival rather than advanced stage as a surrogate endpoint in any RCT. Indeed, others have shown that such conditional means are, in a certain sense, optimal surrogates.^14^ Thus, step (4) involves calculating the predicted mortality at diagnosis stratifying by stage and by the result of the screening test (immediately) prior to diagnosis. Ideally, the same stratification can be done for those in the control arm by retrospectively testing their stored samples. Finally, once a pilot screening programme has been introduced, one should (5) study stage-specific survival stratified by both stage and screening history: screen-detected, interval, or never screened. Additionally, screening programmes should be audited to ensure that individuals with screen-detected cancers are not disadvantaged either in terms of (6) delays in initiating treatment or (7) in terms of the type and quality of treatment received.

The verification outcomes shown in Fig. 1 and discussed in Table 5 are also likely to also be relevant to check assumptions used in health-economic modelling. It is also possible that the health-economic model might require data to support other assumptions on the mechanism of effect of screening or other aspects; and collection and evaluation of data to inform all such assumptions should also be planned in the design and implementation of pilots as far as possible.

## Discussion

Trial-level meta-regression of RCTs is frequently used to justify use of surrogate endpoints in cancer treatment trials. Similar analysis approaches have been assessed for cancer screening trials. However, we identified that meta-analysis trial-level correlation coefficients are not necessarily directly related to the utility of potential surrogate endpoints. Three notable points are raised.

First, a finding of a statistically significant effect from a surrogate endpoint but not from cancer mortality may not contraindicate the surrogate's validity: it may simply indicate that the follow-up period is insufficient to observe the full effect on mortality or that the trial was inadequately powered for mortality.

Second, the correlation between the effect on the surrogate and the effect on mortality, may be far from 1 even for a valid surrogate. Further, when the correlation is very large (as for lung cancer) this is because most advanced stage cancers are fatal and most early-stage cancers are not. In such cases, advanced stage provides a reasonable approximation to cancer mortality. However, for such cancers, death often comes soon after diagnosis, so the advantage of the surrogate endpoint may be small.

Third, even when advanced stage is an invalid surrogate, there may be a significant correlation across trials between the effect of screening on mortality and the effect of screening on advanced stage. In hindsight this makes sense. Suppose that screening reduces advanced stage by 20% but has no impact on mortality. Now consider a trial in which, by chance, there is a 35% reduction in advanced cancer in the screening arm. Those extra 15% diagnosed with early stage rather than late stage will have the fatality benefit of being diagnosed (not necessarily by screening) at early stage. Thus, one would expect a reduction in cancer mortality in the screening arm (relative to the control arm) in that trial. Conversely, consider a trial in which, there happened to be no reduction in advanced stage in the screening arm. That will have happened because, in that trial, there were more cancers that would have presented at advanced stage (in the absence of screening) in the screening arm. Hence, we would expect that cancer mortality will be worse in that trial's screening arm. Thus, even though advanced stage is an invalid surrogate, because it is highly prognostic, the reduction in mortality (across trials) will be correlated with the reduction in advanced stage.

Our analysis suggests that different evidence should be used to decide whether to use advanced stage as a surrogate endpoint in a cancer screening trial. Rather than requiring a meta-analysis of previous RCTs that evaluated both mortality and advanced stage, we argued for supplementary empirical evidence to an RCT of a new screening test, that need not come from randomised trials. To this end, we note that in considering a new framework for working with alternative endpoints, it is not the harms and costs of screening that we need to focus on, but the harms and costs of making the wrong decision regarding the adequacy of a surrogate endpoint—that in turn may lead to wrong decisions about continuing or discontinuing research into a particular screening test. We therefore argued for the importance of testing causal assumptions for reliable use of surrogate endpoints, using a new framework. The idea of developing a causal framework for inference has been developed in other fields, particularly epidemiology,^15^ and to evaluate potential surrogate endpoints in vaccine trials.^16^ Causal assumptions are also used implicitly by natural history models used to investigate the health-economics of screening tests and so testing those assumptions, as proposed here, will also serve to increase confidence in such models.

This analysis has several limitations. First, we did not directly consider the role of timing of cancer incidence versus mortality endpoints. Instead, in the simulations we use a fixed fatality and assume that the mortality analysis is based on the same cancers included in the advanced stage analysis. Thus, our mortality endpoint is “cancers diagnosed by Date 1 that resulted in death by Date 2”. (Date 1 could be the same as Date 2 but need not be). For ease of simulation, we simplified the time-to-event structure underlying cancer screening trials by using binary endpoints both for death from cancer and for diagnosis of advanced stage. This simple model cannot be used to study phenomena such as lead-time and length bias that are inherent to survival in screen-detected cancer. Nor can it be used to study the impact of secular trends in treatment efficacy or stage migrations. However, whereas these phenomena are important for the overall evaluation of cancer screening, they do not affect evaluation of an incidence-based surrogate for cancer mortality.

Second, there are unlikely to be perfect surrogates as assumed in the simulations. We did not consider a partial mediation scenario because the limitations of trial-level correlation may be shown by considering 100% and 0% mediation (i.e., scenarios 1 and 3; see results). Further, our focus is on preserving the type I error—we are worried that we might conclude that screening is effective by studying the surrogate instead of cancer-specific mortality, for which the 0% mediation scenario important. We are less concerned, here, about the loss in power stemming from use of an endpoint that is only a partial mediator. Provided screening cannot positively affect late stage without also positively affecting mortality, it is a valid surrogate for demonstrating efficacy (but not necessarily for accepting that screening does not work). Nonetheless, it is likely that there will be residual confounding within stage categories. Thus, even if the entire effect of early detection is mediated through stage shift, we will not observe independence of fatality and screening conditional on stage. However, this will tend to lead to the underestimation of the mortality benefits by using a binary (early/advanced) stage classification. Conversely, if the screening test preferentially detects aggressive cancers (as is likely to be the case for tests based on cell-free DNA), then a surrogate based on stage alone will likely overestimate the benefit on mortality. Nevertheless, provided stage is still predictive of fatality in screen-detectable cancers, a reduction in advanced stage disease should, on average, result in some reduction in mortality.

Third, we did not consider multi-cancer tests specifically, nor choice of endpoint in this context. Using a different definition of advanced stage for different types of cancer makes sense when considering single-site cancer screening. A disadvantage of using a different definition of advanced stage for different cancers is that it may seem arbitrary, especially when seeking a surrogate endpoint for trials of multi-cancer screening tests. Indeed, the NHS-Galleri trial uses stage III + cancer incidence (including all stageable cancer types) as its primary endpoint.^1^ We submit that this choice of endpoint is reasonable, even though it may not be optimal.

Fourth, there are other ways to meta-analyse data to assess trial-level surrogacy that we did not consider explicitly. For instance, some have proposed fitting linear mixed models.^17^ These can suffer from convergence problems due either to small between-trial variability or, as is always the case in cancer screening, few trials.^18^ Thus, despite the existence of unifying approaches^19^^,^^20^ to the evaluation of surrogate endpoints, they are seldom used (at least they are seldom cited) because of the lack of suitable data. While more might be learnt from meta-analysis of individual patient data, such data are not available from most cancer screening trials. Whilst high-quality individual patient-level data, structural nested models, or multistate modelling could, in theory, offer more robust insights, they are rarely feasible due to data availability. Thus, we maintain that meta-analysis does not offer a practical way for assessing trial-level surrogacy in cancer screening. This is both because there are so few trials of any one type of screening due to the huge size (cost and time) required for a reasonable powered RCT and because even with sufficient data, results can be misleading because association does not imply causation.

Finally, we comment on overdiagnosis. This is commonly defined as screen detection of cancer that would not have been diagnosed within the individual's lifetime had they not been screened. Harms due to overdiagnosis are a central issue in evaluation of cancer screening. However, by definition, overdiagnosis will not impact cancer-specific mortality. Nor will it affect advanced stage incidence, unless some overdiagnosed cancers are diagnosed at advanced stage. This is very unlikely unless the individual dies of an unrelated cause within a few years of being screened. Therefore, provided screening is offered to otherwise healthy individuals with adequate life expectancy, overdiagnosis will not materially affect the endpoint of advanced cancer incidence. On the other hand, overdiagnosis can be a fundamental problem if the endpoint is the “proportion of cancers that are diagnosed at advanced stage”. This endpoint is biased because it uses the number of cancers diagnosed in each arm as the denominator, rather than the number of randomised individuals. Since overdiagnosis will inflate the denominator in the screening arm (and thereby decrease the proportion with advanced stage), this endpoint will be an invalid surrogate in the presence of overdiagnosis. For a fuller discussion of general issues to consider in choosing a potential surrogate outcome in cancer screening see Webb et al.^2^ If there are overdiagnosed advanced stage cancers, that would bias results against screening and make the use of advanced stage as a surrogate conservative. If there is misclassification such that the cause of death of some patients with overdiagnosed cancer is incorrectly recorded as from the targeted cancer, this will bias results using the mortality endpoint against screening. We note that overdiagnosis will reduce the observed fatality from cancers labelled as early stage, but that is because they are a mixture of overdiagnosed early-stage cancers with zero fatality and true early-stage cancers with a fatality that is unaffected by other patients having an overdiagnosed cancer. To summarise, whereas overdiagnosis is one of the major harms of screening and means that it is almost never appropriate to use the ‘proportion of cancers that are diagnosed at advanced stage’ as an endpoint, overdiagnosis has no impact on cancer-specific mortality and is highly unlikely to have any impact on the ‘proportion of trial participants with (or the rate of) advanced cancer’.

In conclusion, new ways to evaluate surrogate endpoints for cancer screening tests are needed. Meta-analysis is of little value to evaluate surrogate endpoints for new classes of cancer screening tests because there will be few if any previous screening trials powered for cancer-specific mortality, and they take decades to run. Even when such data are available, statistics based on correlation are inappropriate to synthesise the results. We suggest surrogates should be tested based on causal reasoning with assumptions verified using data. One way to check key surrogate endpoint assumptions of new screening tests would be through the design and analysis of pilot implementation studies, as well as long-term follow-up for mortality endpoints (beyond surrogate endpoints) in RCTs. We maintain that this proposed framework is both necessary and pragmatic. Advanced stage was the focus of the previous reviews and the analysis in this paper. The attraction is partly because it is intuitive to replace one bad event (death from the targeted cancer) by another (diagnosis with advanced cancer). However, our methodology would also apply if a stage-based predictive score was used instead of advanced stage (Table 5). Such a predicted mortality endpoint might be a better surrogate endpoint than advanced stage incidence even if the predictions are not well calibrated because it will increase statistical power/efficiency.^14^

## Contributors

PDS: Conceptualisation, data curation, computer coding and data generation, formal analysis, methodology, writing–original draft, writing–review and editing. ARB: Methodology, writing–review and editing. SWD: Methodology, writing–original draft, writing–review and editing. PDS, ARB and SWD accessed and verified the underlying data.

## Data sharing statement

The data generated in this study may be re-generated using the code in its Supplementary Files.

## Declaration of interests

PDS is the lead statistician on the NHS-Galleri trial and the BEST4 Screening trial both of which are using advanced stage as the primary endpoint. He is a paid member of GRAIL's Scientific Advisory Board. ARB reports grants or contracts with institution outside this work in the past 36 months from NIHR, Cancer Research UK, Breast Cancer Now, MRC, Prostate Cancer UK, Barts Charity, Cepheid, Unicancer France; royalties to institution and self from Cancer Research UK; consulting fees to institution and self from Median Technologies; consulting fees to self from Kings College London; Participation on a data monitoring committee (Imperial College London), and other potential conflicts of interesting arising from roles astrial statistician on the BEST4 study and statistician on other trials or studies related to cancer screening. SWD is a lead statistician on the PROSPECTS and MyPEBS trials both of which are using advanced stage as a surrogate endpoint in breast cancer screening.

## Appendix A Supplementary data

Evidence for surrogates in screening eCMsupplement
