# Supplementary material for: Evaluating the basis for use of advanced stage as a surrogate endpoint for cancer mortality in screening trials: a simulation study of meta-correlation and an alternative framework
Source: eClinicalMedicine. 2026 Apr 1;94:103862. doi: 10.1016/j.eclinm.2026.103862 (PMC13087788; doi:10.1016/j.eclinm.2026.103862)
Supplement: Evidence for surrogates in screening eCMsupplement [file mmc1.docx]

**When is advanced stage a suitable surrogate for cancer mortality in screening trials?**

**Supplementary material**

**S1. Stata code for the simulations**

set obs 240000

qui {

gen n0=runiform(900,105000)

gen rr=cond(mod(_n,4)==1,exp(-runiform(.05,.35)), exp(-runiform(.2,.45)))

gen null=mod(_n,2)==0

replace rr=1 if null==1

gen site = "prostate"

replace n0=runiform(7500,220000) in 1/20000

gen adv_stage=runiform(.008,.035)

gen e_stage=3*adv_stage

gen f_e=.04

gen f_a=.28

replace site = "breast1" in 20001/40000

replace n0=runiform(18000,80000) if site=="breast1"

replace adv_stage=runiform(.005,.015) if site=="breast1"

replace e_stage=adv_stage if site=="breast1"

replace f_e=.1 if site=="breast1"

replace f_a=.4 if site=="breast1"

replace site = "breast" in 200001/220000

replace n0=runiform(18000,80000) if site=="breast"

replace adv_stage=runiform(.004,.01) if site=="breast"

replace e_stage=2*adv_stage if site=="breast"

replace f_e=.1 if site=="breast"

replace f_a=.4 if site=="breast"

replace site = "breast3" in 220001/240000

replace n0=runiform(18000,80000) if site=="breast3"

replace adv_stage=runiform(.003,.0075) if site=="breast3"

replace e_stage=3*adv_stage if site=="breast3"

replace f_e=.1 if site=="breast3"

replace f_a=.4 if site=="breast3"

replace site = "bowel" in 40001/60000

replace n0=runiform(14000,85000) if site=="bowel"

replace adv_stage=runiform(.005,.011) if site=="bowel"

replace e_stage=adv_stage if site=="bowel"

replace f_e=.2 if site=="bowel"

replace f_a=.65 if site=="bowel"

replace site = "lung" in 60001/80000

replace n0=cond(uniform()<.85, runiform(900,27000), ///

runiform(65000,80000)) if site=="lung"

replace adv_stage=runiform(.007,.033) if site=="lung"

replace e_stage=0.5*adv_stage if site=="lung"

replace f_e=.1 if site=="lung"

replace f_a=.9 if site=="lung"

replace site = "ovary" in 80001/100000

replace n0=runiform(9500,155000) if site=="ovary"

replace adv_stage=runiform(.004,.01) if site=="ovary"

replace e_stage=0.4*adv_stage if site=="ovary"

replace f_e=.25 if site=="ovary"

replace f_a=.7 if site=="ovary"

replace site = "prostate short fu" in 100001/120000

replace n0=runiform(9000,225000) if site=="prostate short fu"

replace adv_stage=runiform(.005,.02) if site=="prostate short fu"

replace e_stage=4*adv_stage if site=="prostate short fu"

replace f_e=.015 if site=="prostate short fu"

replace f_a=.1 if site=="prostate short fu"

*replace rr=exp(-runiform(.05,.4)) if site=="prostate short fu" & null==0

replace site = "liver" in 120001/140000

replace n0=runiform(2000,25000) if site=="liver"

replace adv_stage=runiform(.004,.025) if site=="liver"

replace e_stage=0.75*adv_stage if site=="liver"

replace f_e=.85 if site=="liver"

replace f_a=.95 if site=="liver"

replace site = "other" in 140001/200000

replace n0=runiform(2500,75000) if site=="other"

replace adv_stage=runiform(.004,.025) if site=="other"

replace e_stage=runiform(.005,.025) if site=="other"

replace f_e=runiform(.05,.6) if site=="other"

replace f_a=invlogit(logit(f_e)+runiform(1.5,4)) if site=="other"

*Numbers in control arm

gen n3_4 = rbinomial(n0,adv_stage)

gen n1_2 = rbinomial(n0,e_stage)

gen n_d = rbinomial(n3_4,f_a) + rbinomial(n1_2,f_e)

*Numbers in screening arm

gen m3_4 = rbinomial(n0,adv_stage*rr)

gen m1_2 = rbinomial(n0,e_stage+adv_stage*(1-rr))

gen m_shift = rbinomial(n0,adv_stage*(1-rr)) // number down-staged

gen m_d = rbinomial(m3_4,f_a) + rbinomial(m1_2,f_e)

gen m0_d = rbinomial(m3_4+m_shift,f_a) + rbinomial(m1_2-m_shift,f_e)

gen md_c= rbinomial(m3_4 + m1_2, ///

(adv_stage*f_a+e_stage*f_e)/(adv_stage+e_stage))

lab var n3_4 "N adv stage, control"

lab var n1_2 "N early stage, control"

lab var n_d "N deaths, control"

lab var m3_4 "N adv stage, screen"

lab var m1_2 "N early stage, screen"

lab var m_shift "N downstaged"

lab var m_d "N deaths, screen"

lab var m0_d "N deaths, screen with false surrogate"

lab var md_c "N deaths, screen under no assoc"

gen r_as=1-m3_4/n3_4

gen r_m = 1-m_d/n_d

gen r_asn = r_as[_n-1] if !null // NB the obs alternate between rr=1 & rr<1

gen r_mn = r_m[_n-1] if !null // NB the obs alternate between rr=1 & rr<1.

gen rm_c=1-md_c/n_d

gen rm_f=1-m0_d/n_d

gen z_m=(n_d-m_d)/sqrt(n_d+m_d)

gen z_a=(n3_4-m3_4)/sqrt(n3_4+m3_4)

gen z0_m=(n_d-md_c)/sqrt(n_d+md_c)

gen zf_m=(n_d-m0_d)/sqrt(n_d+m0_d)

lab var r_as "RR adv stage"

lab var r_m "RR mort"

lab var r_asn "RR adv stage under null"

lab var r_mn "RR mort under null"

lab var rm_c "RR mort under no assoc"

lab var rm_f "RR mort under false"

lab var z_m "Z mortality"

lab var z_a "Z adv stage"

lab var z0_m "Z mort under no assoc"

lab var zf_m "Z mort under false"

foreach var of varlist r_as r_m r_asn r_mn rm_c rm_f {

gen l`var'=ln(1-`var')

local vname : var lab `var'

lab var l`var' "log `vname'"

}

}

* NB changed to use log_rr in correlations and regressions 25/04/25

table site ///

if !null, command(r(rho): pwcorr lr_as lr_m) ///

command(r(rho): pwcorr lr_asn lr_mn) ///

command(r(rho): pwcorr lr_as lrm_c) ///

command(r(rho): pwcorr lr_as lrm_f) nform(%8.2f)

table (site result) ///

if !null, nformat(%8.3f) command( ///

downstaged=exp(e(b)[1,2]+e(b)[1,1]*(ln(.85)) + invnorm(.975)* ///

sqrt(e(V)[2,2]+e(V)[1,1]*(ln(.85))^2 +2*e(V)[1,2]*(ln(.85)))) ///

upstaged=exp(e(b)[1,2]+e(b)[1,1]*ln(1.05) + invnorm(.025)* ///

sqrt(e(V)[2,2]+e(V)[1,1]*ln(1.05)^2 +2*e(V)[1,2]*ln(1.05))) : ///

reg lr_m lr_as) ///

command( ///

downstaged=exp(e(b)[1,2]+e(b)[1,1]*(ln(.85)) + invnorm(.975)* ///

sqrt(e(V)[2,2]+e(V)[1,1]*(ln(.85))^2 +2*e(V)[1,2]*(ln(.85)))) ///

upstaged=exp(e(b)[1,2]+e(b)[1,1]*ln(1.05) + invnorm(.025)* ///

sqrt(e(V)[2,2]+e(V)[1,1]*ln(1.05)^2 +2*e(V)[1,2]*ln(1.05))) : ///

reg lr_mn lr_asn) ///

command( ///

downstaged=exp(e(b)[1,2]+e(b)[1,1]*(ln(.85)) + invnorm(.975)* ///

sqrt(e(V)[2,2]+e(V)[1,1]*(ln(.85))^2 +2*e(V)[1,2]*(ln(.85)))) ///

upstaged=exp(e(b)[1,2]+e(b)[1,1]*ln(1.05) + invnorm(.025)* ///

sqrt(e(V)[2,2]+e(V)[1,1]*ln(1.05)^2 +2*e(V)[1,2]*ln(1.05))) : ///

reg lrm_c lr_as) ///

command( ///

downstaged=exp(e(b)[1,2]+e(b)[1,1]*(ln(.85)) + invnorm(.975)* ///

sqrt(e(V)[2,2]+e(V)[1,1]*(ln(.85))^2 +2*e(V)[1,2]*(ln(.85)))) ///

upstaged=exp(e(b)[1,2]+e(b)[1,1]*ln(1.05) + invnorm(.025)* ///

sqrt(e(V)[2,2]+e(V)[1,1]*ln(1.05)^2 +2*e(V)[1,2]*ln(1.05))) : ///

reg lrm_f lr_as)

* Added weighted correlations and regressions 12/11/25

* Due to only being able to put weights in once for the table (not separately for each command), we use the same weights for each of the four regressions

table site [aw=1/(1/m_d + 1/n_d)] ///

if !null, command(r(rho): pwcorr lr_as lr_m) ///

command(r(rho): pwcorr lr_asn lr_mn) ///

command(r(rho): pwcorr lr_as lrm_c) ///

command(r(rho): pwcorr lr_as lrm_f) nform(%8.2f)

table (site result) [aw=1/(1/m_d + 1/n_d)] ///

if !null, nformat(%8.3f) command( ///

downstaged=exp(e(b)[1,2]+e(b)[1,1]*(ln(.85)) + invnorm(.975)* ///

sqrt(e(V)[2,2]+e(V)[1,1]*(ln(.85))^2 +2*e(V)[1,2]*(ln(.85)))) ///

upstaged=exp(e(b)[1,2]+e(b)[1,1]*ln(1.05) + invnorm(.025)* ///

sqrt(e(V)[2,2]+e(V)[1,1]*ln(1.05)^2 +2*e(V)[1,2]*ln(1.05))) : ///

reg lr_m lr_as) ///

command( ///

downstaged=exp(e(b)[1,2]+e(b)[1,1]*(ln(.85)) + invnorm(.975)* ///

sqrt(e(V)[2,2]+e(V)[1,1]*(ln(.85))^2 +2*e(V)[1,2]*(ln(.85)))) ///

upstaged=exp(e(b)[1,2]+e(b)[1,1]*ln(1.05) + invnorm(.025)* ///

sqrt(e(V)[2,2]+e(V)[1,1]*ln(1.05)^2 +2*e(V)[1,2]*ln(1.05))) : ///

reg lr_mn lr_asn) ///

command( ///

downstaged=exp(e(b)[1,2]+e(b)[1,1]*(ln(.85)) + invnorm(.975)* ///

sqrt(e(V)[2,2]+e(V)[1,1]*(ln(.85))^2 +2*e(V)[1,2]*(ln(.85)))) ///

upstaged=exp(e(b)[1,2]+e(b)[1,1]*ln(1.05) + invnorm(.025)* ///

sqrt(e(V)[2,2]+e(V)[1,1]*ln(1.05)^2 +2*e(V)[1,2]*ln(1.05))) : ///

reg lrm_c lr_as) ///

command( ///

downstaged=exp(e(b)[1,2]+e(b)[1,1]*(ln(.85)) + invnorm(.975)* ///

sqrt(e(V)[2,2]+e(V)[1,1]*(ln(.85))^2 +2*e(V)[1,2]*(ln(.85)))) ///

upstaged=exp(e(b)[1,2]+e(b)[1,1]*ln(1.05) + invnorm(.025)* ///

sqrt(e(V)[2,2]+e(V)[1,1]*ln(1.05)^2 +2*e(V)[1,2]*ln(1.05))) : ///

reg lrm_f lr_as)

qui gen mort_sig = z_m>invnorm(.975)

qui gen as_sig = z_a>invnorm(.975)

table site if !nul, statistic (mean mort_sig as_sig) nform(%8.2f)

table site if nul, statistic (mean mort_sig as_sig) nform(%8.3f)

table site if !null , command(r(rho): pwcorr lr_as lr_m) ///

command(r(rho): pwcorr lr_as lrm_f) nform(%8.2f)

table site if !null & n0>=70000, command(r(rho): pwcorr lr_as lr_m) ///

command(r(rho): pwcorr lr_as lrm_f) nform(%8.2f)

table site if !null & n0<=25000, command(r(rho): pwcorr lr_as lr_m) ///

command(r(rho): pwcorr lr_as lrm_f) nform(%8.2f)

**S2. Supplementary tables**

| **Table S1.** Pearson correlation of valid and invalid surrogate endpoints with mortality in simulation scenario when screening effects the surrogate | | | | | | |
| --- | --- | --- | --- | --- | --- | --- |
|  | All trials | | N>=70,000 | | N<=25,000 | |
| Cancer site | Valid surrogate | Invalid surrogate | Valid surrogate | Invalid surrogate | Valid surrogate | Invalid surrogate |
| Bowel | 0.75 | 0.44 | 0.78 | 0.34 | 0.72 | 0.54 |
| Breast | 0.58 | 0.32 | 0.63 | 0.27 | 0.55 | 0.38 |
| Liver | 0.60 | 0.58 | - | - | 0.60 | 0.58 |
| Lung | 0.93 | 0.67 | 0.98 | 0.32 | 0.93 | 0.70 |
| Ovary | 0.79 | 0.47 | 0.84 | 0.35 | 0.76 | 0.64 |
| Prostate | 0.70 | 0.17 | 0.80 | 0.12 | 0.51 | 0.30 |
| Prostate (short) | 0.38 | 0.12 | 0.48 | 0.09 | 0.24 | 0.19 |
| All combined | 0.61 | 0.41 | 0.66 | 0.21 | 0.60 | 0.54 |

| **Table S2.** Weighted correlation of valid and invalid surrogate endpoints with mortality in simulation scenario. Weights inversely proportional to the variance of the logarithm of the mortality ratio. | | | |
| --- | --- | --- | --- |
|  | Valid surrogate | | Invalid surrogate |
| Cancer site | Correlation if screening is effective | Correlation under null hypothesis |  |
| Bowel | 0.76 | 0.69 | 0.40 |
| Breast | 0.60 | 0.51 | 0.30 |
| Liver | 0.56 | 0.74 | 0.52 |
| Lung | 0.96 | 0.93 | 0.50 |
| Ovary | 0.82 | 0.77 | 0.39 |
| Prostate | 0.81 | 0.44 | 0.12 |
| Prostate- short follow-up | 0.49 | 0.26 | 0.09 |
| All combined | 0.63 | 0.66 | 0.34 |

| **Table S3.** Upper limit of 95% CI on RR for mortality from the weighted regression analysis when RR for surrogate is 0.85 in simulation scenarios. Weights inversely proportional to the variance of the logarithm of the mortality ratio. | | |
| --- | --- | --- |
| Cancer site | Valid surrogate | Invalid surrogate |
| Bowel | 0.93 | 1.03 |
| Breast | 0.93 | 1.02 |
| Liver | 1.02 | 1.03 |
| Lung | 0.88 | 1.03 |
| Ovary | 0.93 | 1.02 |
| Prostate | 0.91 | 1.01 |
| Prostate- short F-U | 0.92 | 1.01 |
| All | 0.93 | 1.02 |
